# Mint3-mediated L1CAM expression in fibroblasts promotes cancer cell proliferation via integrin α5β1 and tumour growth

**DOI:** 10.1038/oncsis.2017.27

**Published:** 2017-05-15

**Authors:** H J Nakaoka, Z Tanei, T Hara, J S Weng, A Kanamori, T Hayashi, H Sato, A Orimo, K Otsuji, K Tada, T Morikawa, T Sasaki, M Fukayama, M Seiki, Y Murakami, T Sakamoto

**Affiliations:** 1 Division of Molecular Pathology, Department of Cancer Biology, Institute of Medical Science, The University of Tokyo, Shirokanedai, Minato-ku, Tokyo, Japan; 2 Department of Pathology, Graduate School of Medicine, The University of Tokyo, Hongo, Bunkyo-ku, Tokyo, Japan; 3 Division of Cancer Cell Research, Department of Cancer Biology, Institute of Medical Science, The University of Tokyo, Shirokanedai, Minato-ku, Tokyo, Japan; 4 Division of Molecular Virology and Oncology, Cancer Research Institute, Kanazawa University, Kanazawa, Ishikawa, Japan; 5 Department of Molecular Pathogenesis, Juntendo University School of Medicine, Hongo, Bunkyo-ku, Tokyo, Japan; 6 Department of Breast and Endocrine Surgery, The University of Tokyo Hospital, Bunkyo-ku, Tokyo, Japan; 7 Faculty of Medicine, Institute of Medical, Pharmaceutical and Health Sciences, Kanazawa University, Kanazawa, Ishikawa, Japan

## Abstract

Fibroblasts are some of the major cells in tumour tissues that influence tumour progression and drug resistance. However, our understanding on fibroblast-mediated tumour malignancy remains incomplete. Munc18-1-interacting protein 3 (Mint3) is known as an activator of hypoxia-inducible factor-1 (HIF-1) even during normoxia in cancer cells, macrophages and fibroblasts. Although Mint3 promotes ATP production via glycolysis by activating HIF-1 in cancer cells and macrophages, the biological role of Mint3-mediated HIF-1 activation in fibroblasts remains unclear. To address this, we examined whether Mint3 in fibroblasts contributes to tumour growth. Mint3 depletion in mouse embryonic fibroblasts (MEFs) decreased tumour growth of co-injected human breast cancer cells, MDA-MB-231 and epidermoid carcinoma A431 cells in mice. In MEFs, Mint3 also promoted cancer cell proliferation *in vitro* in a cell–cell contact-dependent manner. Mint3-mediated cancer cell proliferation depended on HIF-1, and further gene expression analysis revealed that the cell adhesion molecule, L1 cell adhesion molecule (L1CAM), was induced by Mint3 and HIF-1 in fibroblasts. Mint3-mediated L1CAM expression in fibroblasts stimulated the ERK signalling pathway via integrin α5β1 in cancer cells, and promoted cancer cell proliferation *in vitro* and tumour growth. In cancer-associated fibroblasts (CAFs), knockdown of MT1-MMP, which promotes Mint3-mediated HIF-1 activation, or Mint3 decreased L1CAM expression. As MEFs, CAFs also promoted cancer cell proliferation *in vitro*, and tumour growth via Mint3 and L1CAM. In human breast cancer specimens, the number of fibroblasts expressing L1CAM, Mint3 and MT1-MMP was higher in cancer regions than in adjacent benign regions. In addition, more phospho-ERK1/2-positive cancer cells existed in the peripheral region surrounded by the stroma than in the central region of solid breast cancer nest. Thus, Mint3 in fibroblasts might be a good target for cancer therapy by regulating cancer cell-stromal cell communication.

## Introduction

Tumour tissues comprise not only cancer cells, but also various types of stromal cells. Fibroblasts are some of the major cells in tumour tissues that influence tumour progression and drug resistance. Especially, fibroblasts in tumour tissues referred to as cancer-associated fibroblasts (CAFs) have features different from those of normal fibroblasts (NFs). CAFs are a pool of heterogeneous cells originated from tissue resident fibroblasts and their progenitor cells, and bone marrow-derived cells.^1, 2, 3^ CAFs secrete various types of cytokines and growth factors, and thereby promote tumour growth, drug resistance and metastasis. Increased number of CAFs correlates with poor prognosis of patients with breast cancer.^4^ However, recent studies reported that some types of CAFs can function as tumour suppressors.^5, 6, 7^ Therefore, targeting the molecular mechanisms by which CAFs promote tumour malignancy is required for developing therapeutic strategies against cancer cell-CAF interaction. However, our understanding on CAF-mediated tumour malignancy remains incomplete.

Hypoxia-inducible factor-1 (HIF-1) is the master regulator of gene expression during hypoxia, and consists of a regulatory α subunit (HIF-1α) and a constitutive β subunit. HIF-1 activation in cancer and stromal cells contributes to tumour malignancy.^8, 9^ We recently revealed that Munc18-1-interacting protein 3 (Mint3) activates HIF-1, even during normoxia, in cancer cells and macrophages.^10, 11, 12, 13^ Mint3 binds to and suppresses factor inhibiting HIF-1 (FIH-1), thereby activating HIF-1. Mint3 also requires the membrane-type matrix metalloproteinase MT1-MMP to activate HIF-1.^11, 14^ Mint3 is expressed in many cell types and increases moderately in tumour tissues.^15, 16^ On the other hand, MT1-MMP is not expressed in normal epithelial cells, but in cancer cells, macrophages, fibroblasts and endothelial tip cells.^17, 18, 19^ Thus, Mint3-mediated HIF-1 activation is limited to these cells. HIF-1 promotes the expression of many glycolysis-related genes.^9, 20, 21^ Mint3 depletion decreases HIF-1 activity and thereby suppresses the enhanced glycolysis during normoxia, known as the Warburg effect in cancer cells.^11^ Because macrophages also depend on glycolysis even during normoxia for their ATP production,^22^ Mint3 depleted macrophages defect in ATP production via glycolysis.^10, 23^ Fibroblasts also activate HIF-1 via MT1-MMP and Mint3 during normoxia.^14^ However, in fibroblasts, ATP production depends on mitochondrial oxidative phosphorylation and Mint3 knockout (KO) mouse embryonic fibroblasts (MEFs) show no defect in ATP production.^10^ Thus, the biological role of Mint3-mediated HIF-1 activation in fibroblasts remains unclear. In this study, we addressed whether Mint3 in fibroblasts contributes to tumour growth by co-injection in mice, and co-culture of cancer cells with control or Mint3-depleted fibroblasts.

## Results

### Mint3 in fibroblasts promotes tumour growth

**Figure 1:**
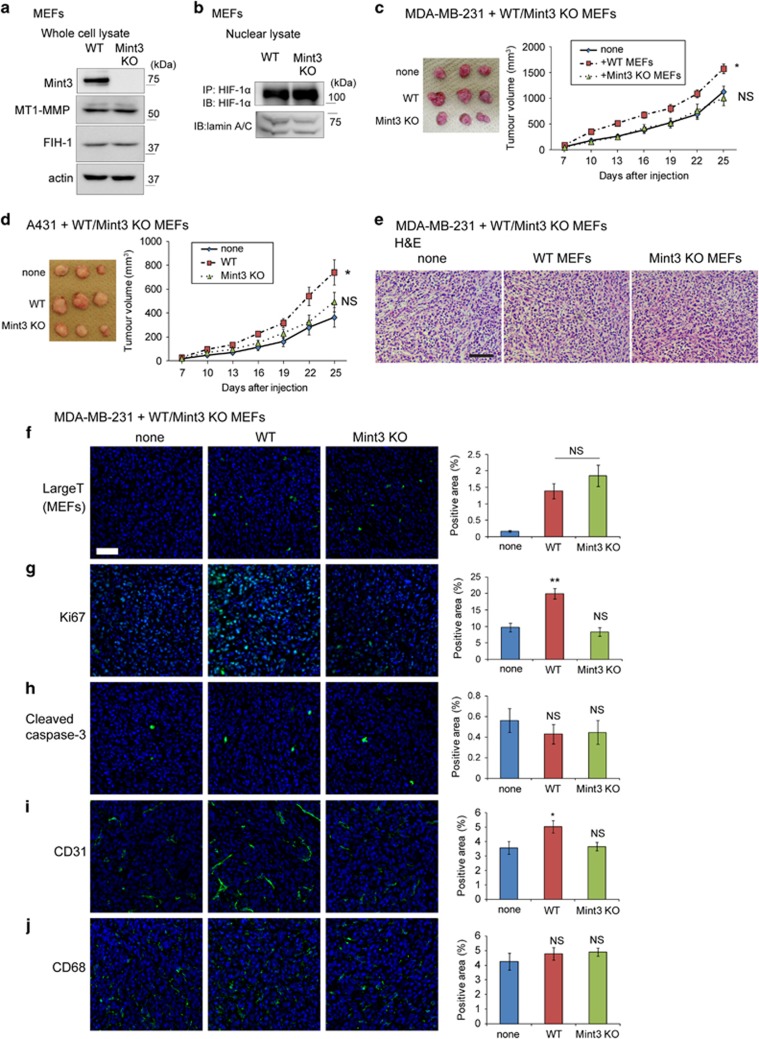
Mint3 in MEFs promotes tumour growth. (**a**) Immunoblot analysis of Mint3, MT1-MMP, FIH-1 and actin in whole-cell lysates from WT and Mint3 KO MEFs. (**b**) Immunoblot analysis of HIF-1α and lamin A/C in nuclear lysates from WT and Mint3 KO MEFs. HIF-1α protein in nuclear lysates was concentrated by immunoprecipitation (IP), followed by immunoblot (IB) for detection. (**c**,**d**) Representative photographs (left panel; day 25) and growth rate (right panel) following subcutaneous injection of MDA-MB-231 (**c**) and A431 cells (**d**), and cells with or without indicated MEFs in immunodeficient mice. (**e**) Haematoxylin and eosin (H&E) staining of tumour tissues derived from MDA-MB-231 cells with or without indicated MEFs at day 10. Scale bar=100 μm. (**f**–**j**) Immunostaining of SV40 large T antigen (**f**), Ki67 (**g**), cleaved caspase 3 (**h**), CD31 (**i**) and CD68 (**j**) in tumour tissues of MDA-MB-231 cells with or without indicated MEFs at day 10 (left panel). Bar=50 μm. Positive areas for each staining were analysed (right panel). In **c** and **d**, the error bars indicate the s.e.m.; *n*=12 from two independent experiments (*n*=6 and *n*=6, respectively); the data shown were analysed using the Mann–Whitney *U*-test. *P<0.05, **P<0.01. In **f**–**i**, the error bars indicate the s.e.m.; *n*=9 from three tumours (three fields per tumour); the data shown were analysed using the Mann–Whitney *U*-test. **P<0.01. NS, not significant.

To clarify the role of Mint3 in fibroblasts, wild type (WT) and Mint3 KO MEFs immortalized by SV40 large T antigen were prepared.^10^ WT and Mint3 KO MEFs expressed MT1-MMP and FIH-1 at comparable levels (Figure 1a). HIF-1α protein levels in nuclear lysates from WT and Mint3 KO MEFs during normoxia were low, but fully detectable and comparable after immunoprecipitation using anti-HIF-1α antibodies (Figure 1b). MEFs were then subcutaneously co-injected with cancer cells in immunodeficient mice. WT MEFs significantly promoted tumour growth of human breast cancer MDA-MB-231 and epidermoid carcinoma A431 cells, while Mint3 KO MEFs did not affect tumour growth of these cells (Figures 1c and d). Thus, these findings indicate that Mint3 depletion in MEFs decreased tumour growth.

To clarify whether Mint3 from fibroblasts affects the tumour condition at the early stage, tumour tissues from mice co-injected with MDA-MB-231 cells and MEFs were analysed at day 10 after injection. Tumours resulting from the co-injection of MDA-MB-231 cells and WT or Mint3 KO MEFs showed no apparent histological difference and had comparable levels of SV40 large T-positive MEFs (Figures 1e and f). Next, the levels of proliferation, apoptosis and stromal markers in tumours were examined. The number of the proliferation marker Ki67-positive cells, but not that of the apoptosis marker cleaved caspase 3-positive cells, was higher in tumours resulting from the co-injection of MDA-MB-231 cells with WT MEFs than in tumours resulting from the co-injection with Mint3 KO MEFs and from MDA-MB-231 cells alone (Figures 1g and h). In addition, Mint3 expression in MEFs slightly increased the number of endothelial marker CD31-positive cells, but not that of the macrophage marker CD68-positive cells, in the tumours from co-injected MDA-MB-231 cells (Figures 1i and j). These results indicated that Mint3 from MEFs promoted cancer cell proliferation and angiogenesis in tumours at an early stage.

### Mint3 from fibroblasts promotes cancer cell proliferation *in vitro* in a cell–cell contact-dependent manner

**Figure 2:**
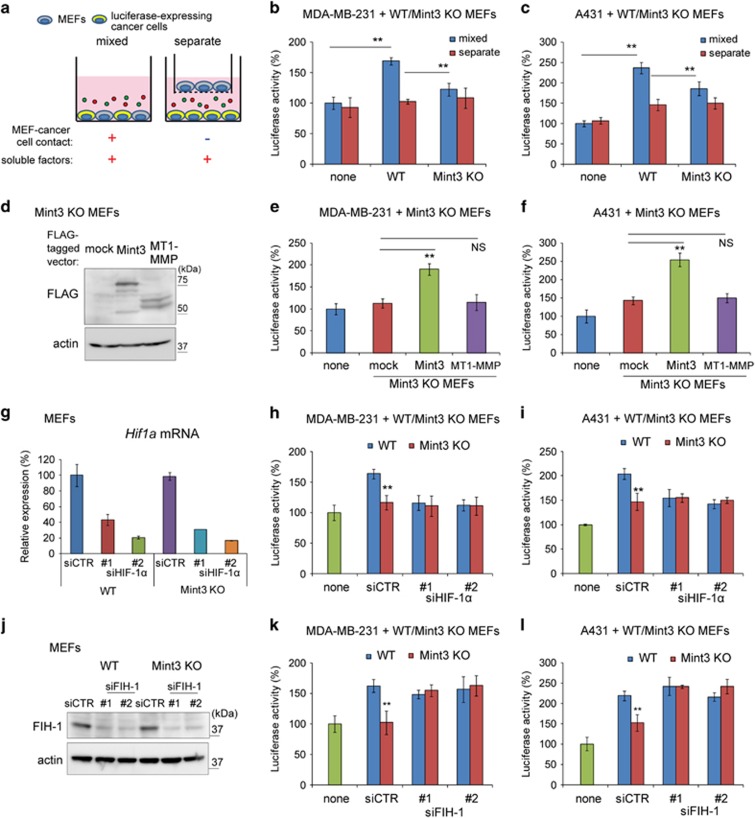
Mint3 in MEFs promotes cancer cell proliferation in a HIF-1-dependent manner. (**a**) Illustration of co-culture experiments. (**b**, **c**) Secreted luciferase activity from GLuc-expressing MDA-MB-231 (**b**) and A431 cells (**c**) co-cultured with MEFs. (**d**) Western blot analysis of mock, FLAG-tagged Mint3 and FLAG-tagged MT1-MMP expression in Mint3 KO MEFs. (**e**, **f**) Secreted luciferase activity from GLuc-expressing MDA-MB-231 (**e**) and A431 cells (**f**) co-cultured with mock, Mint3 or MT1-MMP expressing Mint3 KO MEFs. (**d**) HIF-1α knockdown in MEFs was confirmed by qRT–PCR. (**g**, **h**) Secreted luciferase activity from GLuc-expressing MDA-MB-231 (**e**) and A431 cells (**f**) co-cultured with control or HIF-1α siRNA-treated MEFs. (**i**) FIH-1 knockdown in MEFs was confirmed by western blotting. (**j**, **k**) Secreted luciferase activity from GLuc-expressing MDA-MB-231 (**h**) and A431 cells (**i**) co-cultured with control or FIH-1 siRNA-treated MEFs. In **b**,**c**,**e**–**i**,**k** and **l**, error bars indicate the s.d. (*n*=3). The data were analysed using a *t*-test. *P<0.05, **P<0.01. The data shown are representative of three independent experiments with similar results. NS, not significant; qRT–PCR, quantitative RT–PCR.

Because Mint3 from fibroblasts promoted tumour growth *in vivo*, we next examined whether Mint3 from fibroblasts also promotes cancer cell proliferation *in vitro*. To monitor the proliferation of cancer cells, cancer cells stably expressing secreted-type Gaussia luciferase (GLuc) were co-cultured with MEFs in a mixed condition or a separate condition for 48 h, then luciferase activities in the conditioned media were analysed (Figure 2a). When co-cultured in a mixed condition, WT MEFs significantly increased luciferase activity in the conditioned media of GLuc-expressing MDA-MB-231 and A431 cells when compared with Mint3 KO MEFs (Figures 2b and c, mixed). The luciferase activity in the conditioned medium corresponded to the number of cancer cells, and the number of Mint3 KO MEFs slightly increased compared to that of WT MEFs (Supplementary Figures 1a–c). On the other hand, Mint3 depletion did not affect luciferase activities in conditioned medium from GLuc-expressing MDA-MB-231 and A431 cells when co-cultured in a separate condition (Figures 2b and c, separate). These data indicated that Mint3 from fibroblasts promoted cancer cell proliferation in a cell–cell contact-dependent manner. Exogenous Mint3 expression, but not MT1-MMP expression, in Mint3 KO MEFs restored the proliferation of co-cultured MDA-MB-231 and A431 cells (Figures 2d–f). In turn, Mint3 and MT1-MMP expression in WT MEFs did not strikingly increase cancer cell proliferation (Supplementary Figure 2). Thus, expression levels of endogenous Mint3 in MEFs seem to be necessary and almost sufficient for cancer cell proliferation.

Mint3 activates HIF-1 by suppressing its negative regulator FIH-1.^12^ Thus, we examined whether fibroblast Mint3 promotes cancer cell proliferation via the FIH-1/HIF-1 pathway. HIF-1α knockdown in WT MEFs by small interfering RNA (siRNA) transfection decreased the proliferation of co-cultured MDA-MB-231 and A431 cells to the levels observed when the cells were co-cultured with Mint3 KO MEFs, while that in co-cultures with Mint3 KO MEFs did not decrease further (Figures 2g–i). In addition, FIH-1 knockdown in Mint3 KO MEFs increased the proliferation of co-cultured MDA-MB-231 and A431 cells to the levels observed in co-cultures with WT MEFs (Figures 2j–l). Taken together, Mint3 in fibroblasts promoted cancer cell proliferation via the FIH-1/HIF-1 pathway.

### L1CAM is expressed in fibroblasts in a Mint3-dependent manner

**Figure 3:**
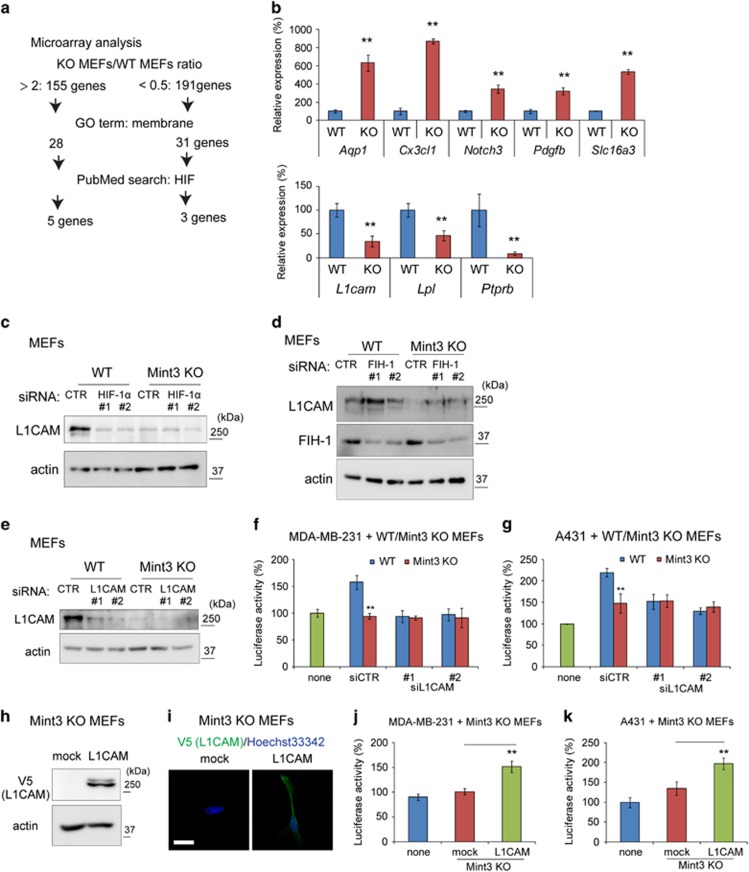
Mint3 induces L1CAM expression in MEFs in a HIF-1-dependent manner. (**a**) Analysis scheme of genes with higher or lower expression in Mint3 KO MEFs. Total RNA of WT and Mint3 KO MEFs from two independent experiments was subjected to microarray analysis. (**b**) qRT–PCR analysis of candidate genes in WT and Mint3 KO MEFs. (**c**) Western blot analysis of L1CAM expression in WT and Mint3 KO MEFs transfected with HIF-1α siRNA. (**d**) Western blot analysis of L1CAM expression in WT and Mint3 KO MEFs transfected with FIH-1 siRNA. (**e**) L1CAM knockdown in MEFs was confirmed by western blotting. (**f**, **g**) Secreted luciferase activity from GLuc-expressing MDA-MB-231 (**f**) and A431 cells (**g**) co-cultured with control or L1CAM siRNA-treated MEFs. (**h**) Western blot analysis of V5-tagged L1CAM expression in Mint3 KO MEFs. (**i**) Immunostaining of V5-tagged L1CAM (green) in Mint3 KO MEFs. Nuclei were counterstained with Hoechst 33342 (blue). Bar=20 μm. (**j**, **k**) Secreted luciferase activity from GLuc-expressing MDA-MB-231 (**c**) and A431 cells (**d**) co-cultured with mock or L1CAM-expressing Mint3 KO MEFs. In **b**, **f**, **g**, **j** and **k**, error bars indicate the s.d. (*n*=3). The data were analysed using a*t*-test. **P<0.01. The data in **b**–**k** shown are representative of three independent experiments with similar results. GO, gene ontology.

Subsequently, to clarify the gene(s) by which the Mint3/FIH-1/HIF-1 pathway in fibroblasts promotes cancer cell proliferation in a cell–cell contact-dependent manner, microarray analyses of gene expression in WT and Mint3 KO MEFs were performed (Figure 3a). The protein-coding genes with twofold higher or 0.5-fold lower expression in Mint3 KO MEFs from two independent experiments were first selected (Supplementary Table 1). Since fibroblast Mint3 promoted cancer cell proliferation in a cell–cell contact-dependent manner, we focused on membrane proteins and chose the genes presenting with ‘membrane’ in the gene ontology term. The genes reported to be associated with HIF were further selected by the keyword search ‘HIF’ in the PubMED database. As a result, five genes with higher expression and three genes with lower expression in Mint3 KO MEFs were identified. The expression of these genes in WT and Mint3 KO MEFs was confirmed by quantitative reverse transcription (RT)–PCR (Figure 3b). Among them, we further focused on *L1CAM*. L1CAM is a type-I transmembrane protein, and L1CAM expression in cancer cells promotes cancer cell proliferation, invasion and metastasis.^24, 25, 26, 27^ Higher expression of L1CAM correlates with poor prognosis of patients with breast, pancreas and squamous cancer,^28, 29, 30^ and L1CAM expression is induced by HIF-1.^31, 32^ However, the role of L1CAM in fibroblasts on cancer progression has not been evaluated yet. Thus, we first examined whether Mint3-dependent L1CAM expression in fibroblasts depends on the FIH-1/HIF-1 pathway. HIF-1α knockdown using siRNA decreased L1CAM expression in WT MEFs to the levels observed in Mint3 KO MEFs (Figure 3c). In turn, FIH-1 knockdown also increased L1CAM expression in Mint3 KO MEFs to the levels of WT MEFs (Figure 3d). Taken together, Mint3 promoted L1CAM expression in fibroblasts via the FIH-1/HIF-1 pathway.

Subsequently, we addressed whether L1CAM contributed to the fibroblast Mint3-mediated cancer cell proliferation. L1CAM knockdown decreased the proliferation of co-cultured MDA-MB-231 and A431 cells to the levels observed when the cells were co-cultured with Mint3 KO MEFs, while that in co-cultures with Mint3 KO MEFs did not decrease further (Figures 3e–g). Next, mock or V5-tagged L1CAM was stably expressed in Mint3 KO MEFs (Figures 3h and i). L1CAM expression in Mint3 KO MEFs increased proliferation of co-cultured MDA-MB-231 and A431 cells (Figures 3j and k). Thus, fibroblast Mint3-mediated cancer cell proliferation can indeed be attributed to L1CAM in fibroblasts.

### Mint3-mediated L1CAM expression in MEFs promotes cancer cell proliferation via integrin α5β1 in cancer cells

**Figure 4:**
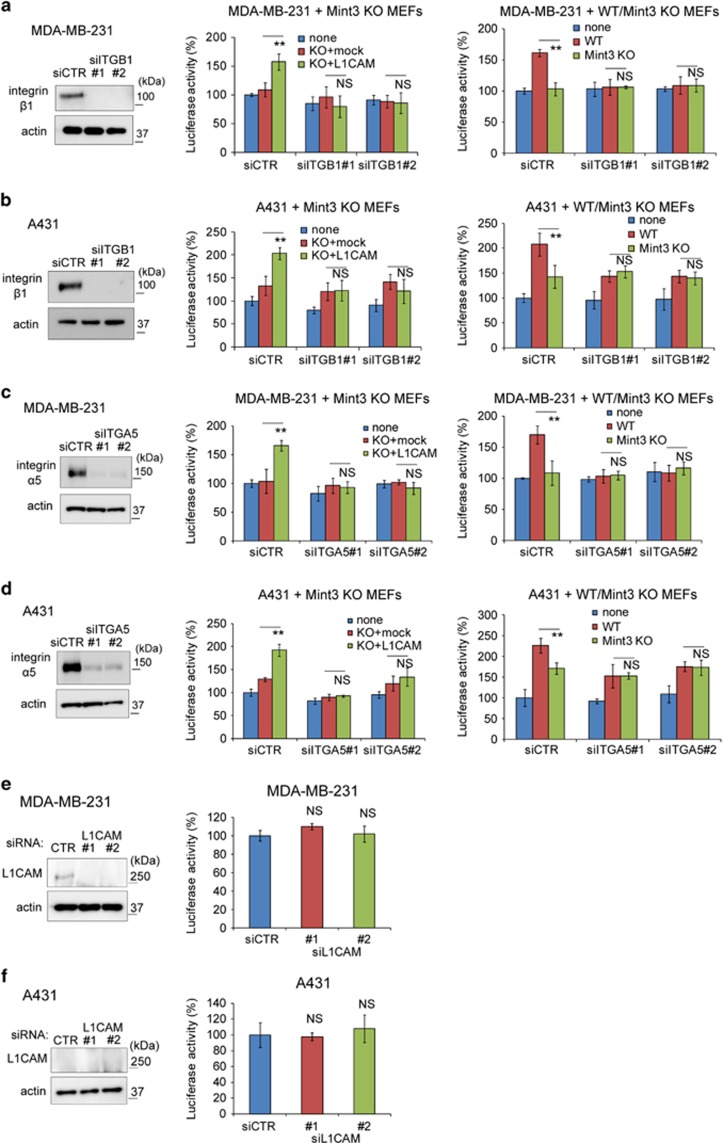
L1CAM in fibroblasts promotes cancer cell proliferation via integrin α5β1. (**a**,**b**) (left panel) Western blot analysis of integrin β1 expression in MDA-MB-231 (**a**) and A431 cells (**b**) treated with control (CTR) or integrin β1 (ITGB1) siRNA. (middle panel) Secreted luciferase activity from siRNA-transfected GLuc-expressing MDA-MB-231 (**a**) and A431 cells (**b**) co-cultured with mock or L1CAM-expressing Mint3 KO MEFs. (right panel) Secreted luciferase activity from siRNA-transfected GLuc-expressing MDA-MB-231 (**a**) and A431 cells (**b**) co-cultured with WT or Mint3 KO MEFs. (**c**, **d**) (left panel) Western blot analysis of integrin α5 expression in MDA-MB-231 (**c**) and A431 cells (**d**) treated with control (CTR) or integrin α5 (ITGA5) siRNA. (middle panel) Secreted luciferase activity from siRNA-transfected GLuc-expressing MDA-MB-231 (**c**) and A431 cells (**d**) co-cultured with mock or L1CAM-expressing Mint3 KO MEFs. (right panel) Secreted luciferase activity from siRNA-transfected GLuc-expressing MDA-MB-231 (**c**) and A431 cells (**d**) co-cultured with WT or Mint3 KO MEFs. (**e**, **f**) (left panel) Western blot analysis of L1CAM expression in MDA-MB-231 (**e**) and A431 cells (**f**) transfected with control (CTR) or L1CAM siRNA. (right panel) Secreted luciferase activity from GLuc-expressing MDA-MB-231 (**e**) and A431 cells (**f**) transfected with control (CTR) or L1CAM siRNA. In **a**–**f**, error bars indicate the s.d. (*n*=3). The data were analysed using a *t*-test. **P<0.01. The data shown in **a**–**f** are representative of three independent experiments with similar results. *P<0.05, **P<0.01. NS, not significant.

L1CAM promotes cell proliferation via integrins.^33, 34^ Thus, we first focused on integrin β1, which is one of the major integrins in cancer cells. Integrin β1 knockdown in MDA-MB-231 and A431 cells did not affect cancer cell proliferation itself, but abrogated Mint3/L1CAM-dependent cancer cell proliferation by MEFs (Figures 4a and b). As partners of integrin β1, integrin α5 and αV are known to bind to L1CAM.^33, 34^ Similarly to integrin β1 knockdown, integrin α5 knockdown in MDA-MB-231 and A431 cells also abrogated Mint3/L1CAM-dependent cancer cell proliferation by MEFs without affecting cancer cell proliferation itself (Figures 4c and d). In contrast, the proliferation of MDA-MB-231 and A431 cells knocked down for integrin αV was decreased, but the cells still responded to L1CAM in Mint3 KO MEFs (Supplementary Figure 3). These data indicated that L1CAM in fibroblasts promoted cancer cell proliferation via integrin α5β1 in cancer cells.

In addition to integrins, L1CAM also binds to L1CAM *in trans* and promotes the proliferation of pancreas cancer cells *in vitro*.^26, 33^ MDA-MB-231 cells, but not A431 cells, expressed L1CAM at a detectable level, although L1CAM knockdown did not affect proliferation in these cells (Figures 4e and f, and Supplementary Figure 4). Integrin α5β1 depletion did not affect the proliferation of MDA-MB-231 and A431 cells (Figures 4a–d). Thus, L1CAM-L1CAM and L1CAM-integrin α5β1 interaction between cancer cells are not likely to be involved in the proliferation of MDA-MB-231 and A431 cells at least *in vitro*.

### Mint3-mediated L1CAM expression in MEFs activates the ERK signalling in cancer cells

**Figure 5:**
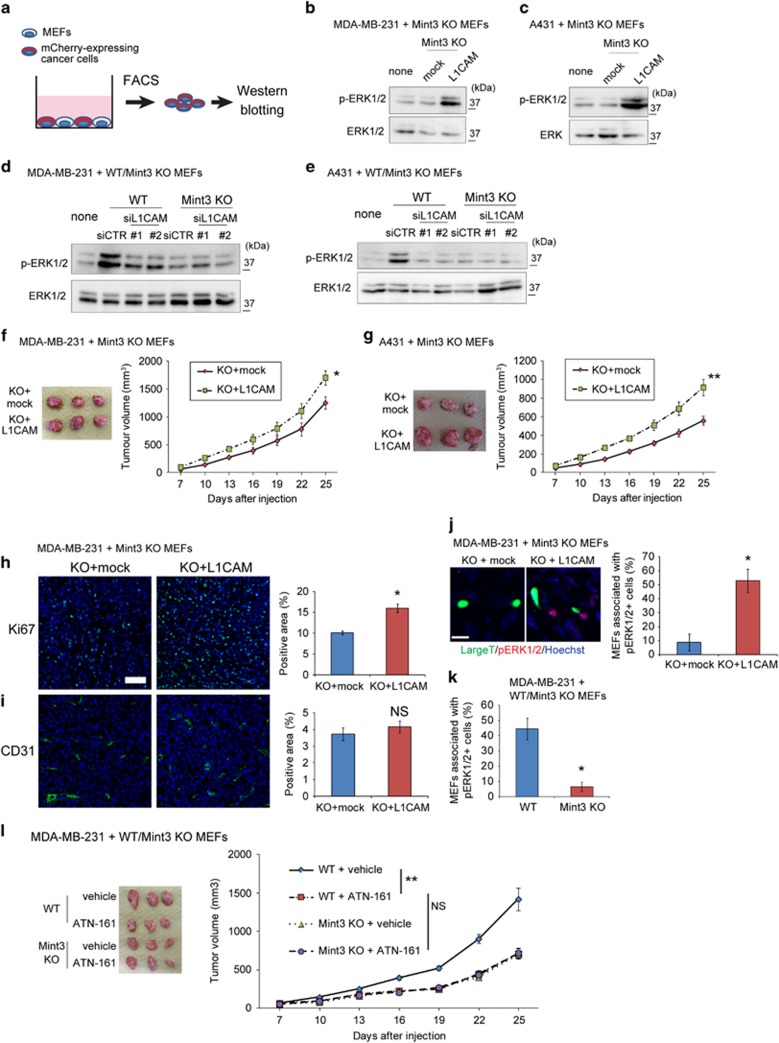
L1CAM in fibroblasts promotes ERK signalling in cancer cells. (**a**) Schematic illustration of the isolation and analysis of mCherry-expressing cancer cells co-cultured with MEFs. (**b**, **c**) Western blot analysis of phospho-ERK1/2 and ERK1/2 in mCherry-expressing MDA-MB-231 (**b**) and A431 cells (**c**) co-cultured with mock or L1CAM-expressing Mint3 KO MEFs. (**d**, **e**) Western blot analysis of phospho-ERK1/2 and ERK1/2 in mCherry-expressing MDA-MB-231 (**d**) and A431 cells (**e**) co-cultured with WT or Mint3 KO MEFs transfected with control (CTR) or L1CAM siRNA. (**f**,**g**) Representative photographs (left panel; day 25) and growth rate (right panel) following subcutaneous implantation of MDA-MB-231 (**f**) and A431 cells (**g**) with mock or L1CAM-expressing Mint3 KO MEFs in immunodeficient mice. (**h**, **i**) Immunostaining of Ki67 (**h**) and CD31 (**i**) in tumour tissues of MDA-MB-231 cells with or without indicated MEFs at day 10 (left panel). Bar=50 μm. Positive areas for each staining were analysed (right panel). (**j**) Representative photo of SV40 large T antigen (green) and phospho-ERK1/2 (red) expression in tumour tissues of MDA-MB-231 cells with mock or L1CAM-expressing Mint3 KO MEFs at day 10 (left panel). The ratio of MEFs associated with phospho-ERK1/2-positive cells in tumour tissues of MDA-MB-231 cells with indicated MEFs was analysed (right panel). (**k**) The ratio of MEFs associated with phospho-ERK1/2-positive cells in tumour tissues of MDA-MB-231 cells with WT or Mint3 KO MEFs was analysed. (**l**) Representative photograph (left panel; day 25) and growth rate (right panel) following subcutaneous injection of MDA-MB-231 cells with indicated MEFs in immunodeficient mice. Mice were subjected to intraperitoneal injection of vehicle or the integrin α5β1 inhibitor ATN-161 (2 mg/kg body weight) every 3 days from day 4 after tumour inoculation. The data shown in **b**–**e** are representative of three independent experiments with similar results. In **f**, **g** and **l**, the error bars indicate the s.e.m.; *n*=12 from two independent experiments (*n*=6 and *n*=6, respectively); the data shown were analysed by using the Mann–Whitney *U*-test. *P<0.05, **P<0.01. In **h** and **i**, the error bars indicate the s.e.m.; *n*=9 from three tumours (three fields per tumour); the data shown were analysed by using the Mann–Whitney *U*-test. *P<0.05. In **j** and **k**, the error bars indicate the s.d.; *n*=5 from five tumours (average of five fields per tumour); the data shown were analysed by using the Mann–Whitney *U*-test. *P<0.05. NS, not significant.

Activated integrins by L1CAM promotes cell proliferation via the ERK signalling.^33, 34^ Thus, mCherry-expressing MDA-MB-231 and A431 cells co-cultured with mock or V5-tagged L1CAM-expressing Mint3 KO MEFs were collected by fluorescence-activated cell sorting, and ERK1/2 phosphorylation levels in these cells were analysed by western blotting (Figure 5a). L1CAM expression in Mint3 KO MEFs increased phosphorylated ERK1/2 levels in co-cultured MAD-MB-231 and A431 cells (Figures 5b and c). In turn, L1CAM knockdown in WT MEFs decreased ERK1/2 phosphorylation levels in MDA-MB-231 and A431 cells to the levels observed in cells co-cultured with Mint3 KO MEFs (Figures 5d and e). Taken together, Mint3/L1CAM in fibroblasts activates the ERK signalling in co-cultured cancer cells.

### L1CAM expression in Mint3 KO MEFs increases tumour growth

We further examined whether L1CAM expression in Mint3 KO MEFs restores tumour growth of co-injected cancer cells. L1CAM-expressing Mint3 KO MEFs significantly increased tumour growth of co-injected MDA-MB-231 and A431 cells compared with mock expressing cells (Figures 5f and g), indicating that fibroblast Mint3 promoted tumour growth at least in part by inducing L1CAM expression. Subsequently, tumour tissues of MDA-MB-231 cells and MEFs at day 10 after injection were analysed. L1CAM expression in Mint3 KO MEFs increased the levels of Ki67, but not those of other markers, including CD31, in the tumour tissues (Figures 5h and i, and Supplementary Figure 5). Thus, L1CAM expression in Mint3 KO MEFs partially restored the proliferation of cancer cells, but did not affect angiogenesis *in vivo*.

Next, we analysed fibroblast-mediated ERK1/2 phosphorylation *in vivo*. L1CAM expression in Mint3 KO MEFs significantly increased the ratio of MEFs associated with phospho-ERK1/2-positive cells in the tumour (Figure 5j). Mint3 expression in MEFs also increased the ratio of MEFs associated with phospho-ERK1/2-positive cells in the tumour (Figure 5k). Thus, fibroblast Mint3/L1CAM increased the levels of phospho-ERK1/2-positive cells around fibroblasts *in vivo*. These results prompted us to examine whether the integrin α5β1 inhibitor, ATN-161,^35, 36^ hampers fibroblast Mint3-mediated tumour growth. ATN-161 administration decreased the ratio of MEFs associated with phospho-ERK1/2-positive cells in the tumour (Supplementary Figure 6), and the tumour growth of MDA-MB-231 cells and WT MEFs; meanwhile, ATN-161 administration did not further decrease the tumour growth of MDA-MB-231 cells and Mint3 KO MEFs (Figure 5l). Taken together, integrin α5β1 was essential for tumour growth mediated by Mint3 in fibroblasts.

### Mint3-mediated L1CAM expression in CAFs promotes tumour growth

**Figure 6:**
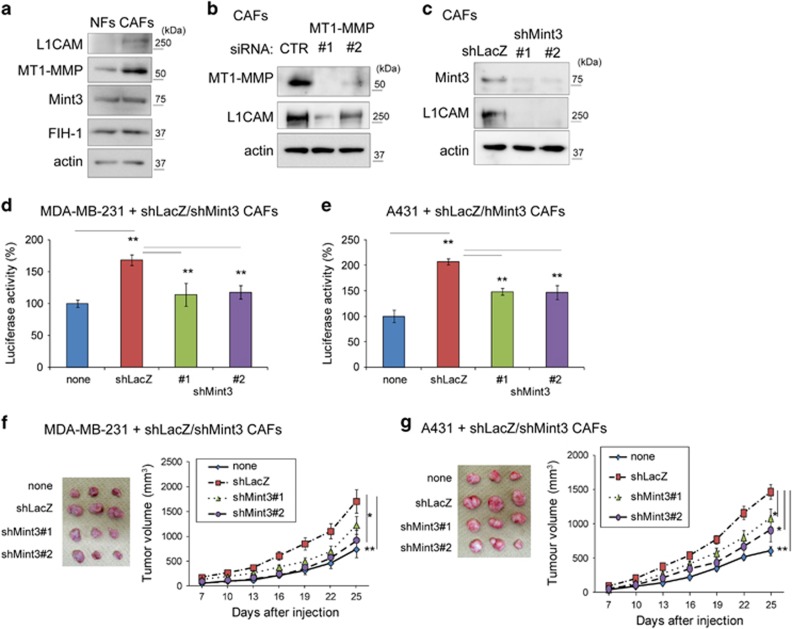
Mint3 in CAFs promotes cancer cell proliferation and tumour growth. (**a**) Western blot analysis of Mint3, L1CAM, MT1-MMP and FIH-1 expression in NFs and CAFs. (**b**) Western blot analysis of L1CAM and MT1-MMP expression in CAFs transfected with control (CTR) or MT1-MMP siRNA. (**c**) Western blot analysis of L1CAM and Mint3 expression in control (shLacZ) or Mint3 knockdown (shMint3) CAFs. (**d**, **e**) Secreted luciferase activity from GLuc-expressing MDA-MB-231 (**d**) and A431 cells (**e**) co-cultured with control or Mint3 knockdown CAFs. (**f**, **g**) Representative photographs (left panel; day 25) and growth rate (right panel) following subcutaneous injection of MDA-MB-231 (**f**) and A431 cells (**g**) with or without indicated CAFs in immunodeficient mice. In **d** and **e**, error bars indicate the s.d. (*n*=3). The data were analysed using a *t*-test. **P<0.01. The data shown in **a**–**e** are representative of three independent experiments with similar results. In **f** and **g**, error bars indicate the s.e.m.; *n*=12 from two independent experiments (*n*=6 and *n*=6, respectively); the data shown were analysed using the Mann–Whitney *U*-test. *P<0.05, **P<0.01.

Next, we investigated whether Mint3 in human CAFs also controls L1CAM expression and tumour growth as observed in MEFs. First, expression levels of Mint3 and its related proteins were examined in NFs and CAFs isolated from normal and human breast cancer tissues from the same patient.^37, 38^ Mint3 and FIH-1 were expressed at comparable levels in NFs and CAFs, but L1CAM and MT1-MMP expression levels increased in CAFs (Figure 6a). MT1-MMP is indispensable for Mint3-mediated HIF-1 activation in cancer cells, macrophages and fibroblasts.^11, 14^ When MT1-MMP was transiently depleted by siRNA, L1CAM levels decreased in CAFs (Figure 6b). L1CAM levels also decreased in Mint3-depleted (shMint3) CAFs compared with control (shLacZ) cells (Figure 6c). Thus, the Mint3/MT1-MMP axis promoted L1CAM expression in CAFs.

Then, control and Mint3-depleted CAFs were co-cultured with MDA-MB-231 and A431 cells. Mint3 depletion in CAFs decreased the proliferation of co-cultured cancer cells as observed in Mint3 KO MEFs (Figures 6d and e). Mint3-depleted CAFs also decreased tumour growth of co-injected MDA-MB-231 and A431 cells when compared with control CAFs (Figures 6f and g).

**Figure 7:**
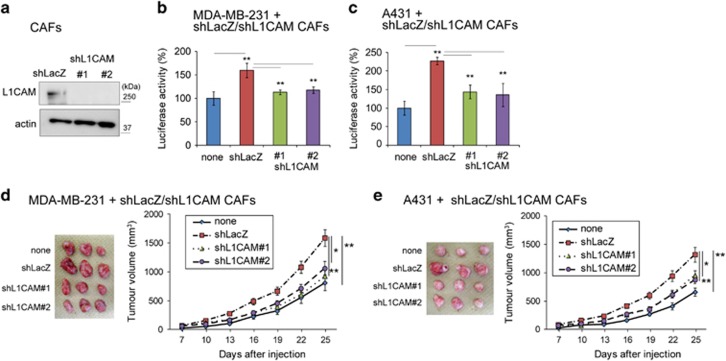
L1CAM in CAFs promotes cancer cell proliferation and tumour growth. (**a**) Western blot analysis of L1CAM expression in control (shLacZ) or L1CAM knockdown (shL1CAM) CAFs. (**b**, **c**) Secreted luciferase activity from GLuc-expressing MDA-MB-231 (**d**) and A431 cells (**e**) co-cultured with control or L1CAM knockdown CAFs. (**d**, **e**) Representative photographs (left panel; day 25) and growth rate (right panel) following subcutaneous injection of MDA-MB-231 (**d**) and A431 cells (**e**) with or without indicated CAFs in immunodeficient mice. In **b** and **c**, error bars indicate the s.d. (*n*=3). The data were analysed using a *t*-test. **P<0.01. The data shown in **a**–**c** are representative of three independent experiments with similar results. In **d** and **e**, the error bars indicate the s.e.m.; *n*=12 from two independent experiments (*n*=6 and *n*=6, respectively); the data shown were analysed using the Mann–Whitney *U*-test. *P<0.05, **P<0.01.

Subsequently, L1CAM-depleted (shL1CAM) CAFs were prepared to examine whether L1CAM in CAFs is also involved in tumour growth (Figure 7a). L1CAM-depleted CAFs decreased *in vitro* proliferation and tumour growth of co-cultured/co-injected MDA-MB-231 and A431 cells, similarly to Mint3-depleted CAFs (Figures 7b–e). Taken together, both Mint3 and L1CAM in CAFs promoted cancer cell proliferation and tumour growth.

### L1CAM, Mint3 and MT1-MMP expression is higher in fibroblasts from breast cancer regions than in those from adjacent non-tumour regions

**Figure 8:**
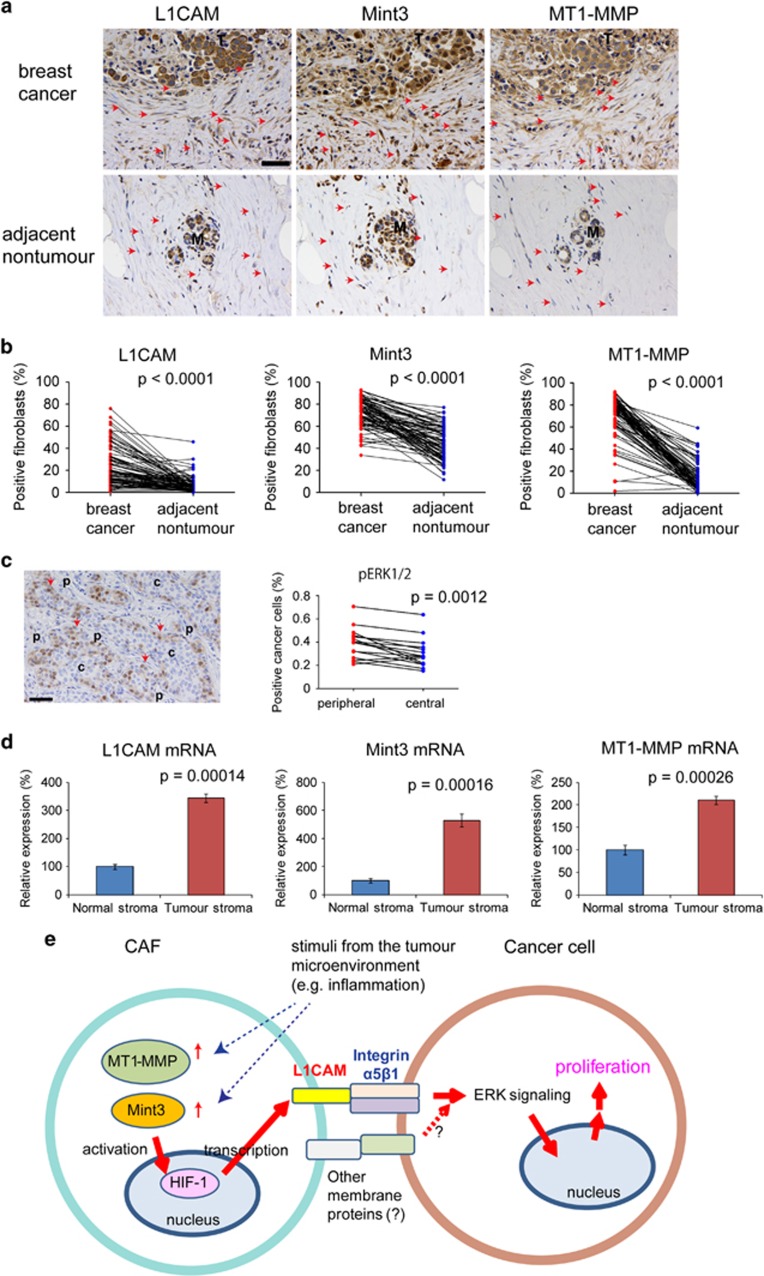
The expression of Mint3, MT1-MMP and L1CAM is higher in fibroblasts from breast cancer tissues. (**a**) Immunostaining of L1CAM, Mint3 and MT1-MMP in breast cancer and adjacent non-tumour regions of the same specimen. Arrows indicate fibroblasts. Bar indicates 50 μm. (**b**) Expression analysis of Mint3, MT1-MMP and L1CAM in fibroblasts from breast cancer and adjacent non-tumour regions. *n*=87. The data were analysed using the paired *t*-test. (**c**) (left) Immunostaining of phospho-ERK1/2 in solid breast cancer. (right) Expression analysis of phospho-ERK1/2 in cancer cells from peripheral and central regions of solid breast cancer. *n*=15. The data were analysed by using the paired *t*-test. (**d**) Expression analysis of Mint3, MT1-MMP and L1CAM mRNA in stromata of breast cancer (*n*=53) and normal regions (*n*=6). The data were analysed by using the Mann–Whitney *U*-test. (**e**) Schematic illustration on how Mint3-mediated L1CAM expression in CAFs promotes cancer cell proliferation. Increased expression of MT1-MMP induces Mint3-mediated HIF-1 activation and thereby promotes L1CAM expression in CAFs. Integrin α5β1 in cancer cells is stimulated by L1CAM in CAFs and activates the ERK signalling, resulting in cancer cell proliferation. c, central; M, mammary gland; p, peripheral; T, tumour.

Finally, the expression of L1CAM, Mint3 and MT1-MMP in human invasive breast cancer specimens was analysed by immunohistochemistry. All three molecules were mainly expressed in cancer cells. However, when we focused on fibroblasts, the number of fibroblasts expressing L1CAM, Mint3 and MT1-MMP was higher in cancer regions than that in adjacent non-tumour regions (Figures 8a and b). In addition, more phospho-ERK1/2-positive cancer cells existed in the peripheral region surrounded by stroma than in the central region of solid breast cancer nest (Figure 8c). We also confirmed higher mRNA levels of L1CAM, Mint3 and MT1-MMP in breast cancer stroma than in normal stroma, using a previously reported public data set (Figure 8d).^39^ These results implied that the tumour microenvironment influenced the expression of L1CAM, Mint3 and MT1-MMP in fibroblasts.

## Discussion

In this study, we showed that Mint3-mediated L1CAM expression in fibroblasts promoted tumour growth. Mint3 activated HIF-1 and thereby induced L1CAM expression in fibroblasts. Induced L1CAM in fibroblasts activated the ERK signalling via integrin α5β1 in cancer cells, resulting in cancer cell proliferation and tumour growth (Figure 8e). In human breast cancer specimens, the number of fibroblasts expressing MT1-MMP was higher in tumour tissues than in normal tissues. MT1-MMP expression is induced by inflammatory cytokines, TGF-β and collagen,^40, 41, 42, 43^ which are characteristic factors of the tumour microenvironment.^44, 45, 46^ Mint3 was also expressed at higher levels in fibroblasts from human breast cancer tissues, although isolated CAFs expressed Mint3 at a level similar to that of NFs. We confirmed that inflammatory cytokines such as TNF-α and interleukin-1β increased Mint3 expression in CAFs (Supplementary Figure 7). Thus, inflammatory conditions might increase Mint3 expression in fibroblasts of tumour tissues and higher MT1-MMP/Mint3 expression might induce L1CAM expression in fibroblasts of human breast cancer tissues.

Many types of cancer cells express L1CAM.^24, 25, 26, 27^ Although MDA-MB-231 expressed L1CAM, L1CAM-L1CAM and L1CAM-integrin α5β1 interactions between cancer cells were not involved in the proliferation of MDA-MB-231 cells. Thus, co-stimulation via other membrane protein(s) might be necessary for a directional proliferation signalling from fibroblasts to cancer cells by L1CAM-integrin α5β1 interaction.

Mint3 depletion in MEFs decreased Ki67-positive and CD31-positive cells in tumours. In turn, L1CAM expression in Mint3 KO MEFs partially restored the levels of Ki67-positive cells, but did not change those of CD31-positive cells. Thus, fibroblast Mint3 might contribute to cancer cell proliferation in the tumour via L1CAM and other angiogenic factors such as VEGFA, which is a representative HIF-1 target gene^8, 9^ and decreased in Mint3 KO MEFs (Supplementary Figure 8). Because the integrin α5β1 inhibitor ATN-161 can inhibit angiogenesis,^35, 36^ we never exclude the possibility that ATN-161 administration might decrease the tumour growth of MDA-MB-231 cells and WT MEFs by inhibiting both L1CAM-mediated proliferation and angiogenesis.

CAFs in tumour tissues are thought to be a pool of heterogeneous cells that play tumour-promoting and tumour-suppressive roles.^1, 2, 3, 5^ Although the net function of CAFs is thought to be tumour promoting in many types of cancer, CAF depletion rather worsens the prognosis in mouse models of pancreatic cancer.^6, 7^ In this study, Mint3/L1CAM depletion in CAFs decreased tumour growth of co-injected MDA-MB-231 and A431 cells. Therefore, inhibiting the tumour-promoting function of CAFs by targeting the Mint3-L1CAM axis might be preferable rather than eliminating CAFs themselves.

In this study, we focused on the tumour growth function of Mint3 in fibroblasts, and revealed that Mint3-mediated L1CAM expression in fibroblasts promoted cancer cell proliferation and tumour growth. In our experimental models, depletion of Mint3 or L1CAM in fibroblasts decreased tumour growth of co-injected cancer cells in mice. However, a limited number of cancer cells can interact with fibroblasts directly in actual tumours. Thus, tumour growth induced by Mint3/L1CAM in fibroblasts might localize at the border between cancer and stromal cells. In addition to the tumour growth, CAFs also contribute to cancer invasion and metastasis.^1, 2, 3^ Gene expression analysis between WT and Mint3 KO MEFs showed that expression of several metalloproteinases such as ADAM19 and TLL1 decreased in Mint3 KO MEFs (Supplementary Table 1). In addition, mRNA levels of EphA1, an invasion/metastasis-associated membrane protein,^47, 48^ also decreased in Mint3 KO MEFs. Unfortunately, in this study, we could not examine whether fibroblast Mint3 contributes to metastasis because no metastasis was observed in our xenograft models. Further studies will uncover the role of Mint3 in fibroblasts on other features of fibroblast-mediated tumour malignancy such as drug resistance and invasion/metastasis. Previous studies revealed that Mint3 depletion in cancer cells suppresses tumour growth of various types of cancer.^11, 13, 16^ In addition, this study showed that Mint3 depletion in fibroblasts also suppressed tumour growth of breast cancer and epidermoid carcinoma. Therefore, Mint3 might be a good target for cancer therapy by regulating not only cancer cells, but also cancer cell-stromal cell communication.

## Materials and methods

### Cell culture

The human breast cancer MDA-MB-231 and the epidermoid carcinoma A431 cells were purchased from the American Type Culture Collection (Manassas, VA, USA). WT and Mint3 KO MEFs were prepared as previously described.^10^ NFs and CAFs from the same patient with breast cancer were prepared as previously described.^37, 38^ Cells were cultured in Dulbecco’s Modified Eagle Medium (DMEM; MDA-MB-231, A431, MEFs) or DMEM high-glucose (NFs, CAFs) containing 10% foetal bovine serum, 100 units per ml penicillin, and 100 μg/ml streptomycin (Sigma–Aldrich, St Louis, MO, USA) at 37 °C in humidified incubator with 5% CO_2_. For inflammatory cytokine stimulation, CAFs were treated with vehicle, 10 ng/ml TNF-α (Peprotech, Rocky Hill, NJ, USA) or 10 ng/ml interleukin-1β (Peprotech) for 24 h.

### Vector construction

shRNA sequences used in this study are described in Supplementary Table 2. Targeted gene sequences were subcloned as deoxyribose fragments into pENTR/U6 TOPO (Thermo Fisher Scientific, Waltham, MA, USA) and recombined into the lentivirus vector pLenti6 BLOCKiT. Human *L1CAM* cDNA was obtained from MDA-MB-231 cells by RT–PCR. mCherry cDNA (kindly provided by Dr R Tsien, Howard Hughes Medical Institute, University of California, San Diego, CA, USA) was amplified by PCR. Gaussia luciferase (GLuc) cDNA obtained from pSV40-GLuc vector (New England Biolabs, Ipswich, MA, USA) was amplified by PCR. These fragments were subcloned into pENTR/D-TOPO and recombined into the lentivirus vector pLenti6 as described previously.^49^ Lentiviral vectors were generated and used according to the manufacturer’s instructions.

### Co-culture experiments

mCherry-expressing MDA-MB-231 or A431 cells were seeded in 24-well plates (5 × 10^3^ per well) in triplicate with or without the same number of indicated MEFs. Forty-eight hours after seeding, cells were washed with PBS three times and collected after trypsin treatment. mCherry-positive cancer cells and mCherry-negative MEFs were counted using counting chambers by fluorescence microscopy.

### Microarray analysis

Total RNA was isolated from WT and Mint3 KO MEFs using the RNeasy plus mini kit (Qiagen, Hilden, Germany). Microarray analysis of total RNA was performed by Takara Bio (Shiga, Japan) using SurePrint G3 Mouse GE 8x60K Microarray (Agilent Technologies, Santa Clara, CA, USA).

### RNA isolation, RT and quantitative PCR

Total RNA was isolated from cells using the RNeasy plus mini kit (Qiagen) and subjected to RT using Superscript III (Thermo Fisher Scientific) and random primers. The RT products were then analysed by real-time PCR in a 7500 quantitative PCR system (Applied Biosystems (ABI), Foster City, CA, USA) using SYBR Green PCR Master Mix (ABI) and the specific primers (Supplementary Table 2) as previously described.^49, 50^ Expression levels of individual mRNA were normalized to that of *Actb* mRNA.

### Western blot analysis

Cell lysates were prepared as previously described.^11^ Nuclear lysates were collected using the Nuclear Extract Kit (Active Motif, Carlsbad, CA, USA) according to the manufacturer’s instructions. Lysates were subjected to western blot as previously described,^11^ using the specific antibodies (Supplementary Table 2). To detect HIF-1α protein, nuclear lysates were subjected to immunoprecipitation using anti-HIF-1α antibody (BD Biosciences, San Jose, CA, USA) followed by western blotting as previously described.^14^

### Luciferase assay

GLuc-expressing MDA-MB-231 or A431 cells were seeded in 24-well plates (5 × 10^3^ per well) in triplicate with or without the same number of indicated MEFs or CAFs. For the separate culture, MEFs were seeded on transwell inserts with 0.4 μm pore size filters (BD Biosciences). Forty 8 h after seeding, culture media were replaced by fresh media and cells were cultured for 6 h. Luciferase activity in conditioned medium was measured in a GloMax 20/20 luminometer (Promega, Madison, WI, USA) using the BioLux Gaussia Luciferase Assay Kit (New England Biolabs).

### siRNA knockdown

Knockdown by siRNA was carried out by using Lipofectamine RNAiMAX (Thermo Fisher Scientific) as previously described.^13^ The sequence of the siRNA for each gene is described in Supplementary Table 2.

### Immunostaining

Immunostaining of cells was performed by using specific antibodies (Supplementary Table 2) as previously described.^12^ Cells were counterstained with Hoechst 33342, washed five times with PBS, mounted on slides, and imaged by confocal microscopy (Carl Zeiss, Oberkochen, Germany).

### Frozen sections and immunostaining

Frozen sections of tumour tissues were prepared and subjected to immunostaining using the specific antibodies (Supplementary Table 2) as previously described.^50^ The nuclei were counterstained with Hoechst 33342, and the sections were observed by confocal microscopy (Carl Zeiss). For the histological analysis, frozen sections were cut at 4 μm thick, and fixed with 4% PFA. Haematoxylin and eosin staining of tissues was performed using standard methodologies.

### Immunohistochemistry of breast cancer tissues from patients

Invasive breast cancer tissue sections were collected from patients (*n*=134) who underwent surgical resection at the University of Tokyo Hospital in 2009. Informed consent was obtained from all patients and the study was approved by the Institutional Ethics Review Committee. Of the 134 cases, 18 cases who received chemotherapy before surgery, 24 cases without invasive breast cancer region, 4 cases of recurrence and 1 case of additional resection were excluded for further analyses (clinicopathological findings from these 87 patients are summarized in Supplementary Table S3). For phospho-ERK1/2 staining, 15 cases with solid breast cancer were selected from the 87 cases. Immunohistochemical analysis was performed in formalin fixed paraffin embedded, 4 μm thick sections. The sections were first treated with 0.3% hydrogen peroxide in methanol for 15 min to block endogenous peroxidase activity. After blocking by incubating with 2% BSA, sections were incubated with the specific antibodies (Supplementary Table 2). The linked primary antibody was detected with DAKO Envision kit (Dako, Glostrup, Denmark) according to the manufacturer’s instructions. 3,3′-diaminobenzidine tetrahydrochloride was used as a chromogen, whereas haematoxylin was used as a light counterstain. Immunohistological evaluation was performed through light microscopic observation. For L1CAM, Mint3 and MT1-MMP staining, three high power fields of tumour or non-tumour areas were examined and fibroblasts positive for each antibody were counted. Finally, the mean percentage of positive fibroblasts per case was calculated. For phospho-ERK1/2 staining, three high power fields of tumour areas were examined and cancer cells positive for each antibody were counted. Outermost cancer cells in the cancer nest were considered to exist in the peripheral region. Other cancer cells were considered to exist in the central region. Finally, the mean percentage of positive cancer cells per case was calculated.

### Fluorescence-activated cell sorting

Single cell suspensions were prepared using 40 μm cell strainers (BD Biosciences). The cells were washed with PBS containing 2% FBS and sorted using a FACS Aria (BD Biosciences).

### Tumour growth assay

Experimental protocols were approved by the Animal Care and Use Committees of The Institute of Medical Science, University of Tokyo. The tumorigenicity of cells was examined using 6-week-old female BALB/c nude mice (Clea Japan, Tokyo, Japan). Briefly, 1 × 10^6^ (MDA-MB-231) or 5 × 10^5^ (A431) cells were injected subcutaneously into the dorsal side of the mice with or without the same number of indicated MEFs or CAFs. Subsequently, the implanted tumours were blindly measured with a calliper on the indicated days and their volumes were calculated using the formula *V*=(*L* × *W*^2^)/2, where *V* is the volume (mm^3^), *L* is the biggest tumour diameter (mm) and *W* is the smallest tumour diameter (mm).

### Data set analysis

The microarray data set of mRNA expression in the tumour stroma (*n*=53) and normal stroma (*n*=6) from patients with breast cancer (GSE9014) was analysed. *L1CAM* (average of ID 30157 and 41179), *Mint3/APBA3* (ID 30367) and *MT1-MMP/MMP14* (ID 33658) mRNA levels were normalized to that of *ACTB* mRNA (average of ID 174, 1504, 13974, 32448). These mRNA levels in each stroma were averaged from two to four replicates.

### Statistical analysis

We compared two groups using a two-sided *t*-test, paired *t*-test or the Mann–Whitney *U*-test.
