# Supplementary material for: Mint3-mediated L1CAM expression in fibroblasts promotes cancer cell proliferation via integrin α5β1 and tumour growth
Source: Oncogenesis. 2017 May 15;6(5):e334–. doi: 10.1038/oncsis.2017.27 (PMC5523060; doi:10.1038/oncsis.2017.27)
Supplement: Supplementary Figures and Tables [file oncsis201727x1.pdf]

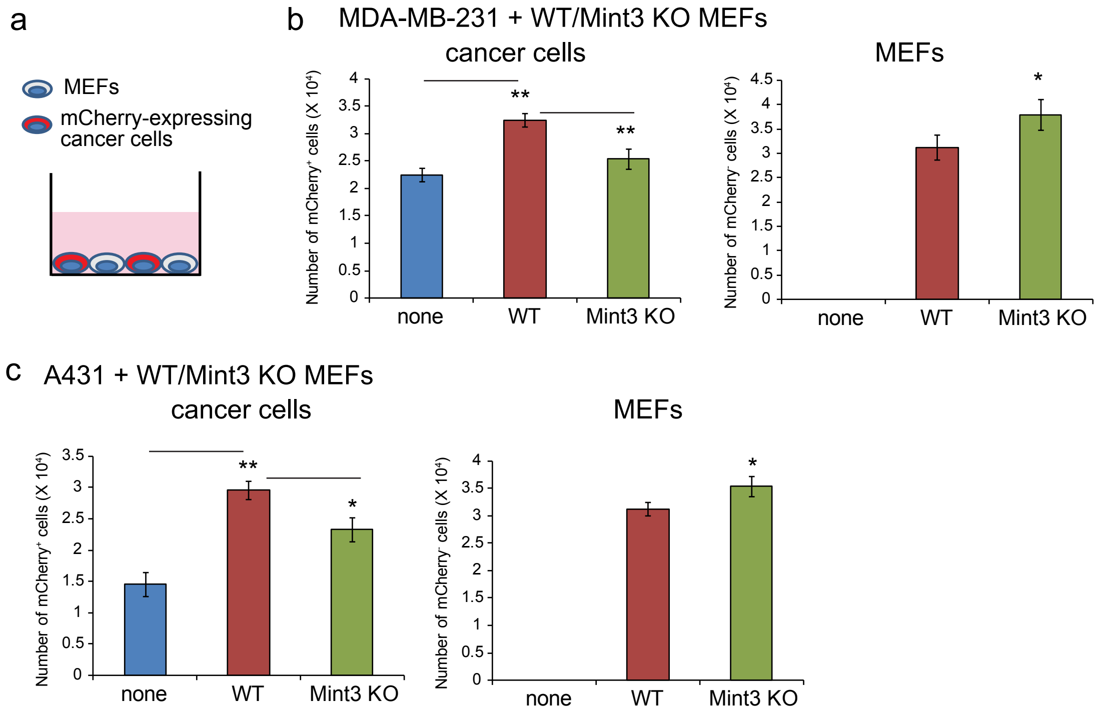


**Supplementary Figure 1. Mint3 KO MEFs defect in promoting cancer cell proliferation.**

(**a**) Illustration of co-culture experiments.

(**b**, **c**) mCherry-expressing MDA-MB-231 (b) and A431 cells (c) were co-cultured with MEFs for 48 h, and mCherry-positive cancer cells (left panel) and mCherry-negative MEFs (right panel) were counted.

In **b** and **c**, error bars indicate the s.d. ($n = 3$). The data were analysed using a t test. *$p < 0.05$, **$p < 0.01$. The data shown in **b-d** are representative of 3 independent experiments with similar results.

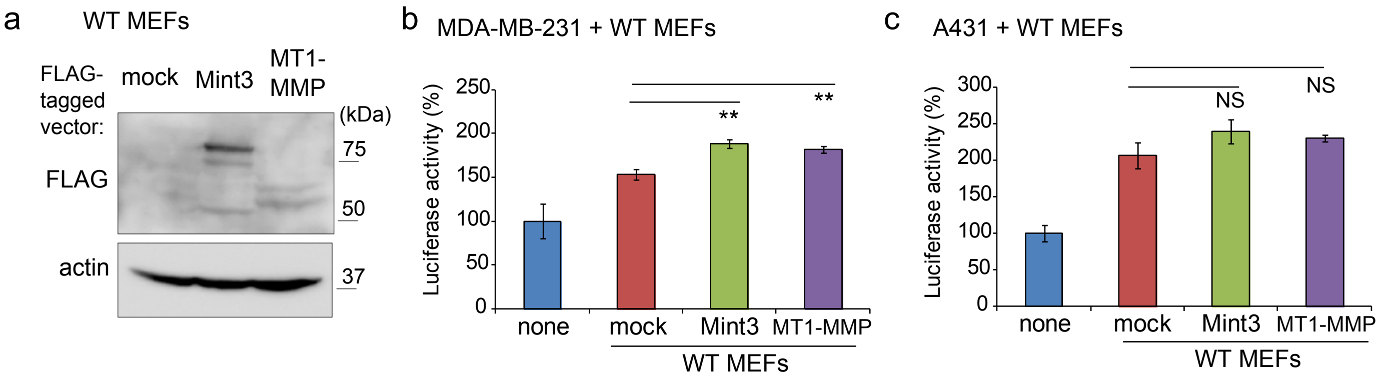


**Supplementary Figure 2. Exogenous expression of Mint3 and MT1-MMP in WT MEFs slightly promotes proliferation of MDA-MB-231 cells.**

(**a**) Western blot analysis of mock, FLAG-tagged Mint3, and FLAG-tagged MT1-MMP expression in WT MEFs.

(**b**, **c**) Secreted luciferase activity from GLuc-expressing MDA-MB-231 (**b**) and A431 cells (**c**) co-cultured with mock, Mint3, or MT1-MMP expressing WT MEFs.

In **b** and **c**, error bars indicate the s.d. ($n = 3$). The data were analysed using a t test. $^{**}p < 0.01$. NS, not significant.

The data shown in **a-c** are representative of 3 independent experiments with similar results.

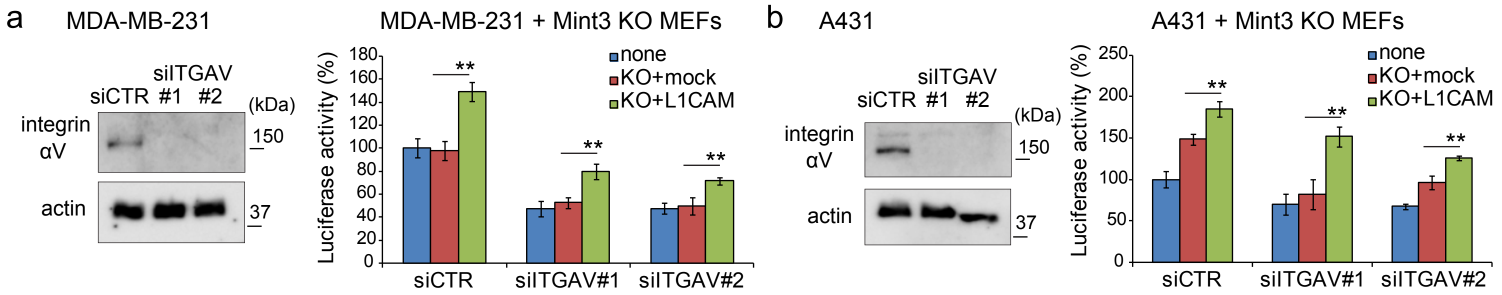


**Supplementary Figure 3. Knockdown of Integrin αV in cancer cells does not affect MEF-mediated proliferation of MDA-MB-231 and A431 cells.**

(**a**, **b**) (left panel) Western blot analysis of integrin expression in MDA-MB-231 (**a**) and A431 cells (**b**) treated with control (CTR) or integrin αV (ITGAV) siRNA. (right panel) Secreted luciferase activity from siRNA-transfected GLuc-expressing MDA-MB-231 (**a**) and A431 cells (**b**) co-cultured with mock or L1CAM expressing Mint3 KO MEFs.

In **a** and **b**, error bars indicate the s.d. ($n = 3$). The data were analysed using a t test. **$p < 0.01$. NS, not significant.

The data are representative of 3 independent experiments with similar results.

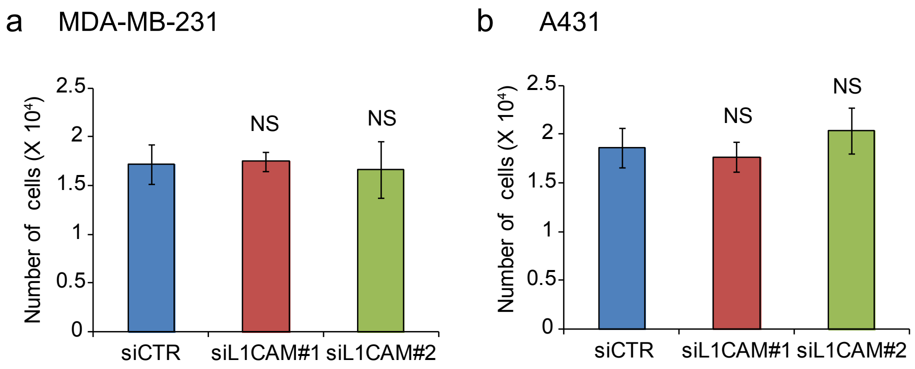


**Supplementary Figure 4. Knockdown of L1CAM does not affect proliferation of MDA-MB-231 and A431 cells.**

(**a**, **b**) Cell number of GLuc-expressing MDA-MB-231 (**a**), A431 cells (**b**) transfected with control (CTR) or L1CAM siRNA.

In **a** and **b**, error bars indicate the s.d. (n = 3). The data were analysed using a t test. NS, not significant.

The data are representative of 3 independent experiments with similar results.

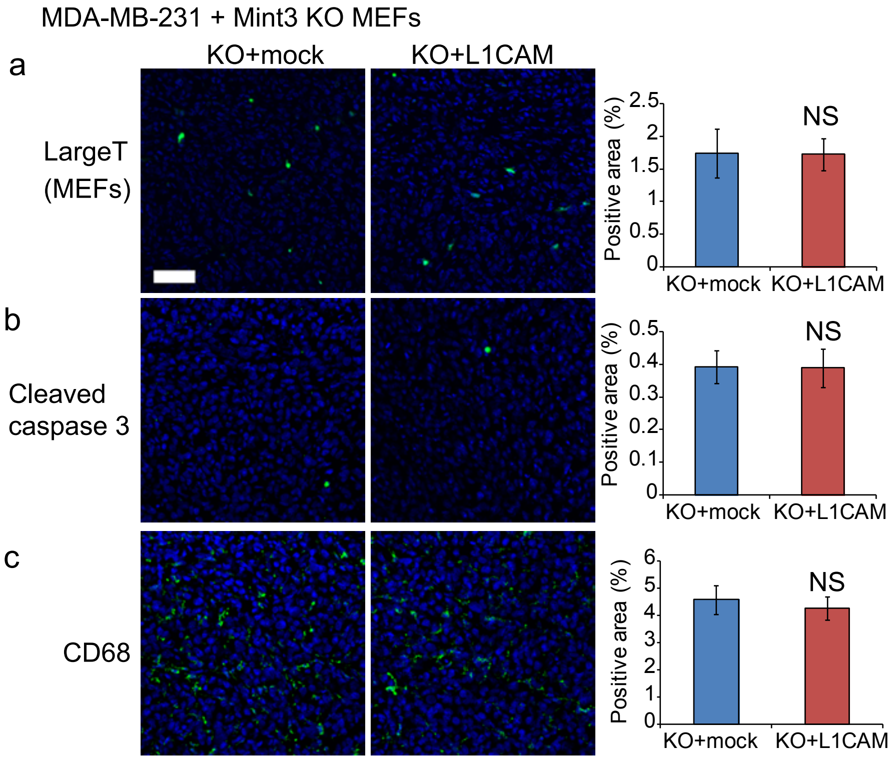


**Supplementary Figure 5. L1CAM expression in Mint3 KO MEFs does not affect the number of MEFs, apoptotic cells, and macrophages in tumour tissues of MDA-MB-231 cells co-cultured with MEFs.**

(**a-c**) Immunostaining of SV40 large T antigen (**a**), cleaved caspase 3 (**b**), and CD68 (c) in tumour tissues of MDA-MB-231 cells cultured with mock or L1CAM expressing Mint3 KO MEFs at day 10 (left panel). Bar = 50 µm. Positive areas for each staining were analysed (right panel).

In **a-c**, the error bars indicate the s.e.m.; n = 9 from 3 tumours (3 fields/tumour); the data shown were analysed by using the Mann–Whitney U-test. NS, not significant.

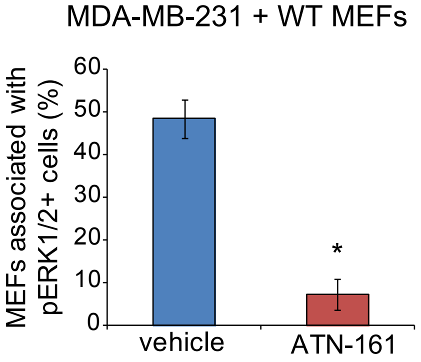


**Supplementary Figure 6. ATN-161 injection decreases the number of MEFs associated with phospho-ERK1/2 positive cells in tumour tissues of MDA-MB-231.**

MDA-MB-231 cells were subcutaneously injected with WT MEFs into immunodeficient mice. At day 10 after tumour injection, mice were administered with vehicle or ATN-161 (2 mg/kg body weight) intraperitoneally, and tumour tissues were collected 4 h after injection followed by immunostaining analysis. The ratio of MEFs associated with phospho-ERK1/2 positive cells in tumour tissues was analysed. The error bars indicate the s.d.; n = 5 from 5 tumours (average of 5 fields/tumour); the data shown were analysed by using the Mann–Whitney U-test. *$p < 0.05$.

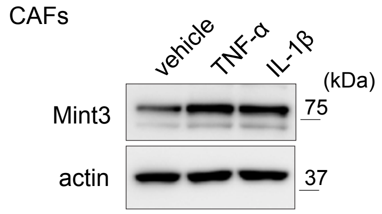


**Supplementary Figure 7. Inflammatory cytokines increase Mint3 expression in CAFs.**

CAFs were treated with vehicle, TNF-α (10 ng/mL), or IL-1β (10 ng/mL) for 24 h followed by western blot analysis.

The data are representative of 3 independent experiments with similar results.

MEFs

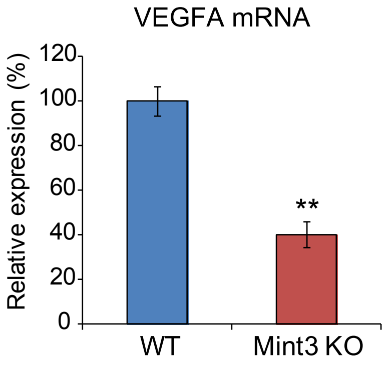


**Supplementary Figure 8. Mint3 depletion decreased VEGFA expression in MEFs.**

RT-qPCR analysis of VEGFA mRNA in WT and Mint3 KO MEFs. Error bars indicate the s.d. ($n = 3$).

The data were analysed by a t test. $^{**}p < 0.05$. The data are representative of 3 independent experiments with similar results.

>2-fold genes (155)

| | |
|---|---|
| 3830403N18Rik | Maf |
| 4930432K21Rik | Mgat3 |
| 4930478M13Rik | Mtss1 |
| 6430553K19Rik | Neto2 |
| 6530409C15Rik | Ngef |
| 6820445E23Rik | Notch3 |
| AA408650 | Npcd |
| Acot1 | Nr1h3 |
| Actn2 | Oas1a |
| Adm2 | Oas1f |
| Adssl1 | Ogdhl |
| Afp | Olfm1 |
| Amz1 | Olfr1431 |
| Anxa8 | Ophn1 |
| Aqp1 | Parp10 |
| Arap3 | Pcdh12 |
| Arhgap9 | Pdcd1lg2 |
| Artn | Pde2a |
| Asphd2 | Pdgfb |
| B4galnt1 | Pdgfra |
| Bace2 | Pik3c2g |
| BC061237 | Pip5kl1 |
| BC068157 | Pkp1 |
| C130030K03Rik | Plek2 |
| C1ql1 | Plekho2 |
| Cadm3 | Ptges |
| Ccl17 | Ptk2b |
| Cd276 | Ptpn18 |
| Cdc42ep5 | Ptpn5 |
| Cdsn | Ptpre |
| Cdyl2 | Qpct |
| Ces1a | Reep1 |
| Ces1g | Rgs17 |
| Chrna4 | Rhox3a |
| Cpa2 | Rhox5 |
| Crabp1 | Rnf130 |
| Crip1 | Rnf183 |
| Ctf1 | Rnls |
| Cx3cl1 | Sardh |
| Cym | Scara3 |
| D930026N18Rik | Serpina10 |
| Dlx4 | Serpinb9f |
| Dnah7a | Sftpd |
| Dnajc22 | Sgcd |
| Dusp2 | Sh2d1b1 |
| Elavl2 | Slc16a3 |
| Enpp2 | Slc35g2 |
| Fam129a | Slc7a2 |
| Fam19a5 | Slit3 |
| Fat4 | Slx |
| Foxred2 | Smpdl3b |
| Gfpt2 | Sp110 |
| Gm10487 | Spn |
| Gm10639 | Srgap3 |
| Gm12349 | Stac2 |
| Gm14625 | Synpo |
| Gm14625 | Sytl2 |
| Gm15085 | Tcf7 |
| Gm1993 | Tmem119 |
| Gm2030 | Trib2 |
| Gm3776 | Trim44 |
| Gm4297 | Trim46 |
| Gm5072 | Ucma |
| Gm5168 | Ugt1a6b |
| Gm9889 | Ulbp1 |
| Grem1 | Ulbp1 |
| Gsc | Vmn1r124 |
| Hapln4 | Vmn1r132 |
| Hmx2 | Vmn2r96 |
| Htr7 | Vps13c |
| Igfbp6 | Xdh |
| Igsf10 | Xlr |
| Irx3 | Zcwpw1 |
| Lama5 | Zfp296 |
| Lef1 | Zswim5 |
| Lmcd1 | |
| LOC552912 | |
| Lrg1 | |
| Luzp4 | |
| Maats1 | |

<0.5-fold genes (191)

| | | |
|---|---|---|
| 1700017I07Rik | Gng13 | Slc24a4 |
| 3830406C13Rik | Gpr126 | Slc26a10 |
| 3830406C13Rik | Grhl1 | Slc35f2 |
| 4930402H24Rik | Gspt2 | Slc38a4 |
| 4930573O21Rik | Hal | Slc6a15 |
| 5830416P10Rik | Hoxa1 | Snap91 |
| 9430014F16Rik | Hspa1a | Snca |
| 9630025H16Rik | Igsf5 | Speer4b |
| A930028N01Rik | Il3ra | Spint1 |
| Abcb9 | Irak3 | St3gal6 |
| Ablim1 | Jakmip2 | Stxbp5l |
| Acap1 | Kcnf1 | Stxbp6 |
| Adam19 | Kcnf1 | Syce2 |
| Adamtsl3 | Klhl13 | Tbx20 |
| Adcy3 | Klhl32 | Tex19.1 |
| Ahrr | Klk1b5 | Tll1 |
| Akip1 | Krt75 | Tmem141 |
| Aldh1a1 | Krtap1-4 | Tmem246 |
| Aldh1a7 | L1cam | Trim71 |
| Aldh1b1 | Lca5l | Tspan13 |
| Arhgap36 | Lce1i | Tspan7 |
| As3mt | Lct | Tspyl5 |
| Asgr1 | Ldb2 | Ttbk1 |
| Atf7ip2 | Lin7a | Ubash3a |
| Atp2b2 | Lmo1 | Ubqlnl |
| Atp6v0a4 | LOC101055644 | Vat1l |
| Avpr1a | LOC101056168 | Wdr78 |
| AY074887 | Lonrf3 | Xk |
| B4galt4 | Lphn2 | Zfp169 |
| Bex1 | Lpl | Zfp518a |
| C530008M17Rik | Lrp2 | Zfp65 |
| Cd33 | Lypd4 | |
| Cd6 | Lypd6 | |
| Cdh13 | Macrod2 | |
| Cdkn1c | Mageh1 | |
| Chd7 | Map2 | |
| Chst10 | March1 | |
| Cnn1 | Mark1 | |
| Cnrip1 | Mid1 | |
| Coch | Mpzl2 | |
| Col2a1 | Msl3l2 | |
| Creld1 | Msrb2 | |
| Cspg4 | Myo5b | |
| Cst7 | Naa11 | |
| Cth | Ndn | |
| Cyp2ab1 | Nefm | |
| Dach1 | Olfr173 | |
| Dcn | Olfr677 | |
| Dlk1 | Olfr868 | |
| Dync1i1 | Olfr969 | |
| Dysf | Parm1 | |
| E2f6 | Pcdh17 | |
| Eda | Pdgfd | |
| Egln3 | Pdlim3 | |
| Eif4g3 | Penk | |
| Elmod1 | Perp | |
| Eml2 | Pla2g2d | |
| Epas1 | Plac8 | |
| Epha1 | Plac9a | |
| Erdr1 | Plagl1 | |
| Etv3l | Plxdc2 | |
| Fabp12 | Polr1b | |
| Fam115c | Pomc | |
| Fam131a | Postn | |
| Fam73a | Prl8a9 | |
| Fam81b | Ptprb | |
| Fbxl7 | Ptprz1 | |
| Fgf14 | Rab38 | |
| Fign | Rbp4 | |
| Frrs1l | Renbp | |
| Gabra2 | Ret | |
| Gabra3 | Robo1 | |
| Galnt3 | Rragb | |
| Gas6 | Rundc3b | |
| Ggn | Scml2 | |
| Glipr1 | Sema6d | |
| Gm10276 | Sfrp2 | |
| Gm2795 | Sgsm1 | |
| Gm6093 | Shroom2 | |
| Gnai1 | Slc16a7 | |

**Supplementary Table 1. Lists of genes with >2-fold and <0.5-fold expression in Mint3 KO MEFs**

**Primer sequences**

| | | |
|---|---|---|
| *Actb* (β-actin) | sense | 5′-agatcaagatcattgctcctcct-3′ |
| | antisense | 5′-acgcagctcagtaacagtcc-3′ |
| *Hif1a* | sense | 5′-catttctcagtcgacacagc-3′ |
| | antisense | 5′-tgtcctgtggtgacttgtcc-3′ |
| *L1cam* | sense | 5′-caccctgaggcattacacct-3′ |
| | antisense | 5′-tgccagtgcagtagcagact-3′ |
| *Aqp1* | sense | 5′-gctgtcatgtacatcatcgcccag-3′ |
| | antisense | 5′-aggtcattgcggccaagtgaat-3′ |
| *Cx3cl1* | sense | 5′-gccattgtcctggagacgac-3′ |
| | antisense | 5′-ccaaggtgatcccaggtgtc-3′ |
| *Notch3* | sense | 5′-ttctcctgtcgttgtctccg-3′ |
| | antisense | 5′-ggcactcatctatgtcactttgg-3′ |
| *Pdgfb* | sense | 5′-gactccgtagatgaagatgg-3′ |
| | antisense | 5′-ttgcactcggcgattacagc-3′ |
| *Slc16a3* | sense | 5′-tcacgggtttctcctacgc-3′ |
| | antisense | 5′-gccaaagcggttcacacac-3′ |
| *Lpl* | sense | 5′-ccaatggaggcactttccag-3′ |
| | antisense | 5′-ccacgtctccgagtcctctc-3′ |
| *Ptprb* | sense | 5′-tctgcagacagaaagctagc-3′ |
| | antisense | 5′-tagaaggtagttggagtctg-3′ |
| *Vegfa* | sense | 5′-agagcagaagtcccatgaag-3′ |
| | antisense | 5′-ctggtagacatccatgaacttg-3′ |

**shRNA sequences**

| | |
|---|---|
| shLacZ | 5′-gcuacacaaaucagcgauuucgaaaaaucgcugauuuguguag-3′ |
| shMint3#1 | 5′-gaugauggcgguggugacgggacgaaucccgucaccaccgccauca-3′ |
| shMint3#2 | 5′-gcgguucuugguccuguaugacgaaucauacaggaccaagaaccgc-3′ |
| shL1CAM#1 | 5′-gcugcuuugccagcaauaagccgaagcuuauugcuggcaaagcagc-3′ |
| shL1CAM#2 | 5′-ggaugaacagcaagaucuugccgaagcaagaucuugcuguucaucc-3′ |

**siRNA sequences**

| | |
|---|---|
| control siRNA | 5′-gaucuacuggucugccuaa-3′ |
| mouse HIF-1α (#1) | 5′-caagcaacugucauauaua-3′ |
| mouse HIF-1α (#2) | 5′-cuuugauguggauagcgau-3′ |
| mouse FIH-1 (#1) | 5′-ccauaaguucuuauacuau-3′ |
| mouse FIH-1 (#2) | 5′-guauugcacgcugcacuua-3′ |
| mouse L1CAM (#1) | 5′-caaugacacuggacgcuau-3′ |
| mouse L1CAM (#2) | 5′-caguaguucugccuugcaa-3′ |
| human integrin β1 (#1) | 5′-cuguucuuuggauacuagu-3′ |
| human integrin β1 (#2) | 5′-cuuacucaaugaaagacga-3′ |
| human integrin α5 (#1) | 5′-cagauaacuucacccgaau-3′ |
| human integrin α5 (#2) | 5′-cacuuuccaugcccagaau-3′ |
| human integrin αV (#1) | 5′-cuuuacugcugauagugcu-3′ |
| human integrin αV (#2) | 5′-cacauugguuaccacuaau-3′ |
| human MT1-MMP (#1) | 5′-gguucuggcgggugaggaa-3′ |
| human MT1-MMP (#2) | 5′-gauggauacccaaugccca-3′ |
| human L1CAM (#1) | 5′-cacacaaccugaccgaucu-3′ |
| human L1CAM (#2) | 5′-ccaagauccugacugcgga-3′ |

**Antibodies (Western blot)**

| antibody | dilution |
|---|---|
| anti-V5 mouse antibody (R960-25; Thermo Fisher Scientific) | 1:1000 |
| anti-actin mouse antibody (MAB1501; Merck Millipore) | 1:1000 |
| anti-Erk1/2 rabbit antibody (#4695; Cell Signaling Technology,) | 1:1000 |
| anti-phospho-Erk1/2 (Thr202/Tyr204) rabbit antibody (#4370; Cell Signaling Technology) | 1:1000 |
| anti-integrin α5 mouse antibody (51-9001996; BD Biosciences) | 1:1000 |
| anti-integrin αV rabbit antibody (ab124968; Abcam) | 1:500 |
| anti-integrin β1 mouse antibody (51-9001995; BD Biosciences) | 1:1000 |
| anti-Mint3 mouse antibody (611380; BD Biosciences) | 1:1000 |
| anti-MT1-MMP mouse antibody (MAB3328; Merck Millipore) | 1:1000 |
| anti-laminA/C mouse antibody (612162; BD Biosciences) | 1:1000 |
| anti-HIF-1α mouse antibody (H1alpha67; Abcam) | 1:1000 |
| Anti-FLAG rabbit antibody (F7425; Sigma) | 1:1000 |
| anti-L1CAM goat antibody (SC-1508; Santa Cruz Biotechnology) | 1:250 |
| anti-FIH-1 goat antibody (SC-26219; Santa Cruz Biotechnology) | 1:250 |

**Antibodies (immunostaining, cell)**

| Antibody | dilution |
|---|---|
| anti-V5 mouse antibody (R960-25; Thermo Fisher Scientific) | 1:500 |
| anti-mouse IgG conjugated to Alexa Fluor 488 goat antibody (A-11029; Thermo Fisher Scientific) | 1: 2000 |

**Antibodies (immunostaining, frozen section)**

| Antibody | dilution |
|---|---|
| rabbit anti-cleaved caspase-3 antibody (#9579; Cell Signaling Technology) | 1:100 |
| rabbit anti-Ki67 antibody (RM-9106-S0; Thermo Fisher Scientific) | 1:400 |
| mouse anti-SV40 large T antigen (ab16879; Abcam) | 1:100 |
| rabbit anti-SV40 large T antigen (#15729; Cell Signaling Technology) | 1:200 |
| anti-phospho-Erk1/2 (Thr202/Tyr204) rabbit antibody (#4370; Cell Signaling Technology) | 1:100 |
| anti-mouse IgG conjugated to Alexa Fluor 488 goat antibody (A-11029; Thermo Fisher Scientific) | 1: 500 |
| anti-rabbit IgG conjugated to Alexa Fluor 546 goat antibody (A-11035; Thermo Fisher Scientific) | 1: 500 |

**Antibodies (immunostaining, paraffin section)**

| Antibody | Antigen retrieval | dilution |
|---|---|---|
| rabbit polyclonal antibody against L1CAM (ab123990; Abcam) | microwave (600 W, 10 min) using 10 mM citrate buffer (pH 6.0) | 1:100 |
| mouse monoclonal antibody against MT1-MMP (MAB3328; Merck Millipore) | microwave (600 W, 10 min) using 10 mM citrate buffer (pH 6.0) | 1:500 |
| rabbit monoclonal antibody against anti-phospho-Erk1/2 (Thr202/Tyr204) (#4370 Cell 1:100Signaling Technology) | microwave (600 W, 10 min) using 10 mM citrate buffer (pH 6.0) | 1:100 |
| rabbit polyclonal antibody against APBA3/Mint3 (HPA045577; Sigma) | microwave (600 W, 10 min) using 100 mM Tris-HCl buffer (pH 9.2) | 1:100 |

Supplementary Table 2. Lists of **detailed material information.**

| Factors | n(%) |
|---|---|
| Average Age (Yr) | 58 (28-85) |
| | |
| Primary tumor | |
| T1b | 13 (14.9%) |
| T1c | 41 (47.1%) |
| T2 | 30 (34.5%) |
| T3 | 1 (1.1%) |
| T4 | 2 (2.3%) |
| | |
| Regional lymph nodes | |
| N0 | 58 (66.7%) |
| N1 | 24 (27.6%) |
| N2 | 4 (4.6%) |
| N3 | 1 (1.1%) |
| | |
| p-Stage | |
| IA | 42 (48.3%) |
| IIA | 28 (32.2%) |
| IIB | 10 (11.5%) |
| IIIA | 5 (5.7%) |
| IIIB | 1 (1.1%) |
| IIIC | 1 (1.1%) |

**Supplementary Table 3 Clinicopathological information from 87 patients with invasive breast cancer**
